# Non-volatile particle emissions from aircraft turbine engines at ground-idle induce oxidative stress in bronchial cells

**DOI:** 10.1038/s42003-019-0332-7

**Published:** 2019-03-05

**Authors:** Hulda R. Jonsdottir, Mathilde Delaval, Zaira Leni, Alejandro Keller, Benjamin T. Brem, Frithjof Siegerist, David Schönenberger, Lukas Durdina, Miriam Elser, Heinz Burtscher, Anthi Liati, Marianne Geiser

**Affiliations:** 1 Institute of Anatomy, University of Bern, 3012 Bern, Switzerland; 2 Institute of Aerosol and Sensor Technology, Swiss University of Applied Sciences and Arts Northwestern Switzerland, 5210 Windisch, Switzerland; 3 Empa, Swiss Federal Laboratories for Materials Science and Technology, Laboratory for Advanced Analytical Technologies, 8600 Dübendorf, Switzerland; 4 SR Technics, 8302 Kloten, Switzerland; 5 Empa, Swiss Federal Laboratories for Materials Science and Technology, Automotive Powertrain Technologies Laboratory, 8600 Dübendorf, Switzerland; 6 Present Address: Laboratory of Atmospheric Chemistry, Paul Scherrer Institute, 5232 Villigen, Switzerland; 7 Present Address: Centre for Aviation, School of Engineering, Zurich University of Applied Sciences, 8401 Winterthur, Switzerland; 8 Present Address: Empa, Swiss Federal Laboratories for Materials Science and Technology, Automotive Powertrain Technologies Laboratory, 8600 Dübendorf, Switzerland

## Abstract

Aircraft emissions contribute to local and global air pollution. Health effects of particulate matter (PM) from aircraft engines are largely unknown, since controlled cell exposures at relevant conditions are challenging. We examined the toxicity of non-volatile PM (nvPM) emissions from a CFM56-7B26 turbofan, the world’s most used aircraft turbine using an unprecedented exposure setup. We combined direct turbine-exhaust sampling under realistic engine operating conditions and the Nano-Aerosol Chamber for In vitro Toxicity to deposit particles onto air–liquid-interface cultures of human bronchial epithelial cells (BEAS-2B) at physiological conditions. We evaluated acute cellular responses after 1-h exposures to diluted exhaust from conventional or alternative fuel combustion. We show that single, short-term exposures to nvPM impair bronchial epithelial cells, and PM from conventional fuel at ground-idle conditions is the most hazardous. Electron microscopy of soot reveals varying reactivity matching the observed cellular responses. Stronger responses at lower mass concentrations suggest that additional metrics are necessary to evaluate health risks of this increasingly important emission source.

## Abstract (web summary)

Hulda R. Jonsdottir et al. examine the effects of non-volatile particulate matter from an aircraft turbine to cultures of human bronchial epithelial cells. They find that short-term exposures to exhaust particles cause cytotoxicity, oxidative stress, and immune infiltration, highlighting the need for further study into the effects of jet fuel-derived pollution.

## Introduction

Emissions from commercial aircraft engines have a substantial impact on both local and global air pollution and are of particular concern for individuals working at airports, as well as local residents (reviewed in ref. ^1^). The steadily increasing demand for commercial air travel and related growth in air traffic indicates an even greater role for aircraft emissions in future global air pollution. At the same time, the number of airport workers will expectedly increase in parallel with the size of nearby residential areas^2^. As of yet, few studies have been conducted on the health effects of particulate matter (PM) from aircraft turbine engines and most of them address self-reported respiratory symptoms in airport workers rather than direct cellular effects from aircraft exhaust exposure^3–6^. Conversely, there is a large body of literature on combustion-generated aerosol from automobile engines consistently concluding that road traffic exhaust has deleterious consequences for human health, for example, increased incidence of cardiovascular disease, asthma exacerbation, and cancer^7–12^. Studies of road traffic PM have shown that both morphological and chemical properties of soot, the main constituent of PM emitted by internal combustion engines, closely relate to particle reactivity^13–15^ and depend on engine operating conditions as well as on fuel type^16–18^. Soot particles emitted by gas turbine engines are, to a large extent, ultrafine, with mobility diameters typically below 100 nm^19–21^ and are generally smaller than those observed in road traffic exhaust^22^. Such small particles deposit with high efficiency in the entire respiratory tract^23,24^ and are supposedly more toxic than larger ones^25–28^, and therefore require special consideration. While substantial information regarding the respiratory toxicology of combustion PM can be obtained from studies on road traffic, particles emitted by aircraft engines have been inadequately investigated. Moreover, although the new international aircraft PM emission standard will use non-volatile PM (nvPM) mass and number as its regulatory metric^29^, a link between health effects and these metrics has not been clearly established. Any adverse health effects caused by combustion-generated particles, primarily soot, are likely associated with physicochemical particle properties, including morphology. However, so far, no clear cause–effect relationship between particle properties and adverse health effects has been documented, although various studies have addressed this issue^30–32^. This applies not only to PM from aircraft turbine engines but also to particles from other combustion sources, for example, diesel, gasoline, and compressed natural gas engines^33,34^. Thus, there is an urgent need for studies linking PM from aircraft turbine engines operating under realistic conditions to health effects. Moreover, as various alternative aviation fuels become more common, it is also important to examine the toxicity of their combustion products, since previous research has not clearly proven the benefits of alternative fuels^35–37^.

Thus, we aimed at elucidating the acute cellular response, when nvPM from a CMF56-7B26 turbofan at different thrust levels, fueled with either conventional Jet A-1 base fuel or an alternative 32% v v^−1^ HEFA (hydroprocessed esters and fatty acid)/base fuel blend, was deposited on human bronchial epithelial cells (BEAS-2B) at air–liquid interface (ALI). For particle deposition under physiological conditions, we used the Nano-Aerosol Chamber for In vitro Toxicity (NACIVT), a portable exposure chamber that can be connected to any aerosol source^38^. Furthermore, we studied the morphological characteristics of soot by transmission electron microscopy (TEM) in an effort to reveal any relationship between these characteristics and observed cellular effects.

Our study demonstrates acute bronchial epithelial cell injury after 1-h exposures to nvPM with the most pronounced response observed after exposure to PM from conventional Jet A-1 base fuel at ground-idle conditions. TEM analysis of soot reveals varying reactivity corresponding to the observed cellular responses. Stronger responses at lower mass concentrations suggest the inclusion of additional metrics for health risks assessment of this increasingly important emission source.

## Results

### Aerosol characterization and nvPM deposition on cells

**Fig. 1:**
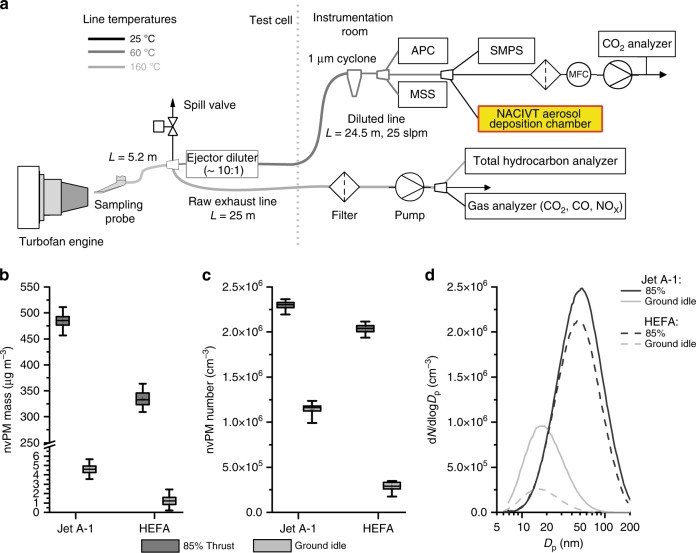
Combustion aerosol sampling from a jet turbine engine and characterization of collected particles. **a** Schematic of experimental setup. The Nano-Aerosol Chamber for In vitro Toxicity (NACIVT) chamber is connected to the diluted particulate matter (PM) sample line in the instrumentation room. APC = AVL Particle Counter Advanced (non-volatile particle number concentration); MSS = AVL micro soot sensor (black carbon mass concentration); SMPS = scanning mobility particle sizer, TSI Model 3938 (particle size distribution). **b** Particle mass concentration for Jet A-1 and HEFA blend. **c** Particle number concentration for Jet A-1 and HEFA blend. **d** Particle size distribution for Jet A-1 and HEFA blend. **b**, **c** Data are presented as box plots, where whiskers represent the 2.5 and the 97.5 percentile. Mean values are indicated with “+”

Combustion aerosol from a commercial turbofan CFM56-7B26 engine, running on Jet A-1 base fuel or 32% HEFA blend (Table 1) at 85% and ground-idle thrust conditions, was collected using a state-of-the-art standardized sampling system (Fig. 1a). The resulting emissions followed commonly observed behavior for turbofans that employ rich quench lean combustors^39^. Particle mass concentrations at the Nano-Aerosol Chamber for In vitro Toxicity (NACIVT) inlet were close to ambient concentrations for ground-idle thrust, for which on average ± SD 4.6 ± 0.7 µg m^−3^ was measured for Jet A-1 and 1.3 ± 0.5 µg m^−3^ for HEFA blend (Fig. 1b and Table 2). At 85% thrust, however, mass concentrations were substantially higher for both fuel types. The average values were 485 ± 14 µg m^−3^ for Jet A-1 and 335 ± 8 µg m^−3^ for HEFA blend. In contrast to mass, particle number concentrations were substantially higher than ambient ones at ground-idle thrust, that is, 1.14 ± 0.04 × 10^6^ cm^−3^ and 0.29 ± 0.10 × 10^6^ cm^−3^ for Jet A-1 and HEFA blend, respectively (Fig. 1c and Table 2). At 85% thrust, number concentrations were higher than at ground-idle, that is, 2.30 ± 0.03 × 10^6^ and 2.04 ± 0.02 × 10^6^ cm^−3^ for the two fuel types. The resulting particle size distributions (Fig. 1d) were unimodal and lognormal, with count median diameters (CMDs) increasing from 18 and 17 nm at ground-idle thrust to 50 and 47 nm at 85% thrust for Jet A-1 and HEFA blend, respectively (Table 2). Our results indicate considerably lower nvPM emissions with HEFA blend, in particular at ground-idle thrust. At this condition, particle mass and number concentrations were reduced by 73 and 75%, respectively; in contrast, at 85% thrust, the reduction was 31% for mass and 11% for number concentrations. The reduction of the nvPM with HEFA blend was likely due to the lower total aromatic content of that blend (higher hydrogen content) compared to Jet A-1 (Table 1). This is consistent with previous studies^39–41^.

**Table 1:** Properties of Jet A-1 base fuel and HEFA fuel blend^a^

Property	Method	Unit	Jet A-1	32% HEFA
Total aromatics	ASTM D 1319	% v v^−1^	18.1	11.3
Naphthalenes	ASTM D 1840	% v v^−1^	0.79	0.53
Hydrogen mass	NMR	% m m^−1^	13.8	14.3
Sulfur content	ASTM D 5453	ppm	490	350
Smoke point	ASTM D 1322	mm	22	24
Density	ASTM D 4052	kg m^−3^	794.8	781.8

*ASTM* American Society for Testing and Materials, *HEFA* hydroprocessed esters and fatty acid, *NMR* nuclear magnetic resonance spectroscopy method comparable to ASTM D7171

^a^32% HEFA blend

**Table 2:** Physical properties of non-volatile exhaust particles

Fuels and thrust levels	Non-volatile particles				
	Mass conc. (µg m^−3^) (SD)	Number conc. (×10^6^ cm^−3^) (SD)	CMD (nm)	estM_dep_^a^ (ng cm^−2^)	estN_dep_^a^ (×10^9^ cm^−2^)
Jet A-1					
85% thrust	485 (14)	2.30 (0.03)	50	432	3.2
Ground-idle	4.6 (0.7)	1.14 (0.04)	18	6.9	2.1
HEFA blend					
85% thrust	335 (8)	2.04 (0.02)	47	311	2.9
Ground-idle	1.3 (0.5)	0.29 (0.01)	17	2.1	0.6

*CMD* count median diameter, *HEFA* hydroprocessed esters and fatty acid, *SD* standard deviation, *estM*_*dep*_ estimated particle mass deposited, *estN*_*dep*_ estimated number of particles deposited on cell cultures

^a^Estimated per hour exposure according to Jeannet et al.^38^

**Fig. 2:**
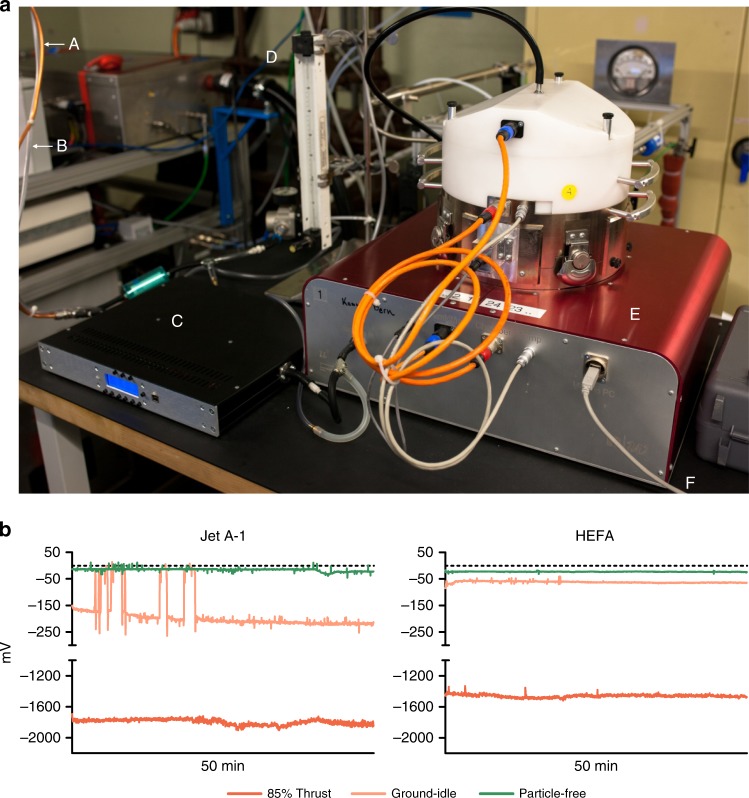
Aerosol exposure setup and particle deposition. **a** NACIVT deposition chamber setup for jet turbine aerosol exposure. A = Exhaust line; B = CO_2_ line; C = low-flow thermodenuder; D = rotameter for regulation of CO_2_ flow into the chamber (5%); E = NACIVT chamber; and F = USB connection to computer for online data collection. **b** Successful deposition of particles on cell cultures. Electrometer data collected within the deposition chamber show distinct precipitation voltage patterns for varying engine thrust levels and fuel types

The aerosol deposition chamber NACIVT (Fig. 2a^38^) was connected to the diluted PM sampling line (shown in Fig. 1a) to expose cell cultures to nvPM continuously for 60 min. Volatile components of the exhaust were removed from the sampling line before entering the NACIVT chamber by a custom-made low-flow thermodenuder^42^. The use of a thermodenuder does not substantially alter the particle size distributions (Supplementary Fig. 1), indicating that the volatile particle mass and number fractions are minimal when aircraft emissions are sampled from the hot core flow of the engine. This is in line with previous observations from aerosol mass spectrometers that studied the emissions of the same engine model^21,42,43^. Successful deposition of nvPM within the NACIVT chamber was confirmed by the electrometer measurements shown in Fig. 2b. The collected data indicated higher particle deposition for Jet A-1 than for HEFA blend at both thrust levels. Furthermore, comparable electrometer voltages (around zero) between fuel types during exposures to filtered exhaust confirmed the successful removal of particles for particle-free control exposures. The estimated deposition on cell cultures for 1 h of exposure, based on Jeannet et al.^38^, showed that at 85% thrust, 432 ng, that is, 3.2 × 10^9^ particles cm^−2^ of cell culture were deposited for Jet A-1 and 311 ng, that is, 2.9 × 10^9^ particles cm^−2^ for HEFA blend (Table 2). At ground-idle, the estimated deposition was considerably lower with 6.9 ng, that is, 2.1 × 10^9^ particles cm^−2^ cell culture for Jet A-1 fuel and 2.1 ng, that is, 0.6 × 10^9^ particles cm^−2^ for HEFA blend. In summary, these data reveal that particle doses applied to the cell cultures were substantially higher at 85% thrust than at ground idle, were higher for Jet A-1 than for HEFA blend, and showed more pronounced differences between fuel types at ground-idle thrust.

### Cellular responses to nvPM exposures

**Fig. 3:**
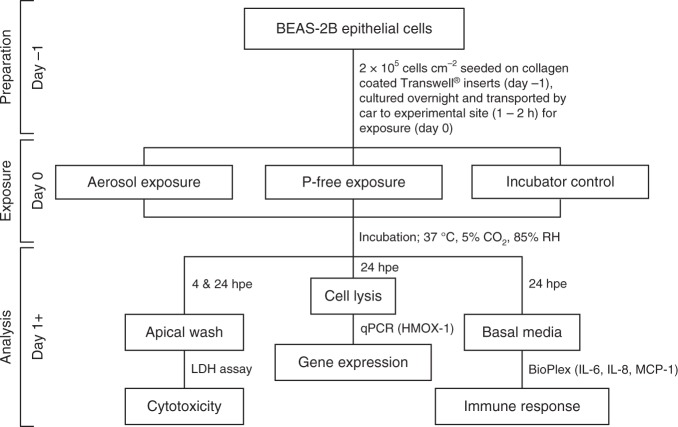
Graphical summary of experimental layout detailing exposure conditions, sampling times, methods, and eventual output. Hpe = hours post exposure; P-free = particle-free

The flowchart in Fig. 3 summarizes the experimental layout for cell exposures, sample collection, and analysis. Quadruplicate cultures of the human bronchial epithelial cell line BEAS-2B at ALI were simultaneously exposed to nvPM for 60 min. Particles were deposited on the apical cell surface under physiological conditions in the NACIVT deposition chamber^38^. To discern effects from deposited particles, we exposed cell cultures to particle-free exhaust by mounting a filter between the PM sample line and the thermodenuder, prior to aerosol entry into the deposition chamber. Incubator control cells were handled the same as all other cell cultures with the exception of any exposure treatment in the NACIVT chamber. Biomarkers for pulmonary response were assessed 24 h after aerosol exposure, thus identifying acute cellular responses.

**Fig. 4:**
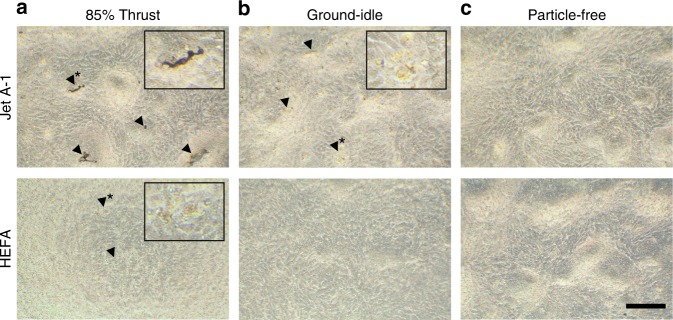
Light microscopy revealed morphologic changes in BEAS-2B cells 24 h after exposure to nvPM. Jet A-1: **a** Black deposits (arrowheads and inset with area at higher magnification*); **b** rounding of cells (arrowheads and inset with area at higher magnification*). HEFA blend: **a** Rounding of cells (arrowheads and inset inset with area at higher magnification*). **c** No morphological changes observed after exposure to filtered exhaust for both fuel types. Scale bar, 200 µm

**Fig. 5:**
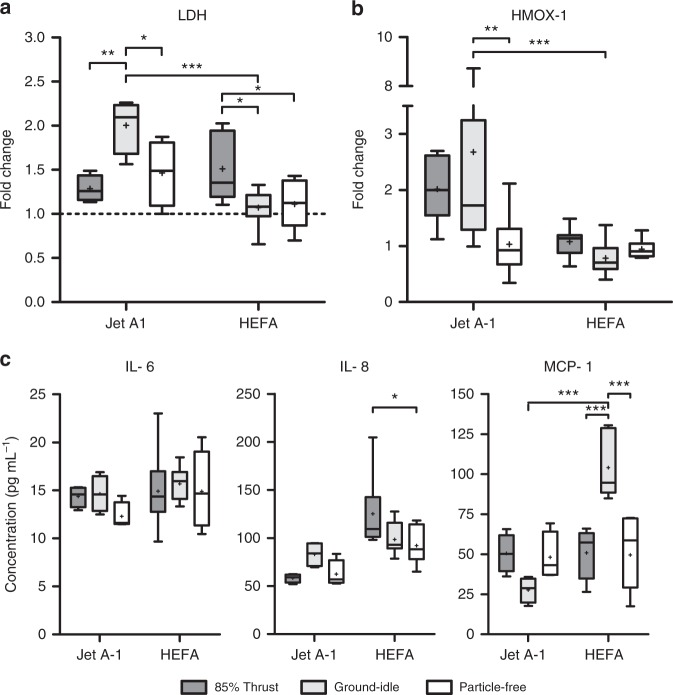
Exposure of epithelial cells to nvPM from Jet A-1 induces LDH release and oxidative stress, while HEFA blend causes increased cytokine release. **a** Release of LDH into apical medium presented as fold change over incubator control (dashed line). **b** Expression of HMOX-1 in cells presented as fold change over incubator control (dashed line). **c** Release of interleukin-6 (IL-6), IL-8, and monocyte chemotactic protein 1 (MCP-1) into basal medium. Data are presented as box plots, where whiskers represent the 2.5 and the 97.5 percentile. Mean values are indicated with “+”. (*n* = 2–4 cultures for Jet A-1, *n* = 4–8 cultures for HEFA blend). Statistical significance was assessed using a non-matching two-way analysis of variance (ANOVA) with Bonferroni post-tests: *p < 0.05, ** *p* < 0.01, and ***p < 0.001

To evaluate acute cytotoxicity, we both assessed the epithelial layer by light microscopy (LM) and quantified the release of lactate dehydrogenase (LDH) by damaged cells into the apical compartment. LM evaluation, shown in Fig. 4, revealed rounding of cells after exposure to nvPM at 85% thrust from both fuel types (Fig. 4a, enlarged area), but at ground-idle only in Jet A-1-exposed cells (Fig. 4b, enlarged area). Particle deposits were only visible after exposure to 85% thrust with Jet A-1 (Fig. 4a, black arrowheads). Measurements of LDH release, shown in Fig. 5a, corroborated LM assessments and revealed a significant increase of cytotoxicity in cells exposed to nvPM at ground-idle with Jet A-1 compared to HEFA blend (*p* < 0.0001). Interestingly, there was limited cytotoxicity in cells exposed at 85% thrust for Jet A-1. However, in contrast to Jet A-1, cytotoxicity after exposure to HEFA blend nvPM was significantly higher in cells exposed at 85% thrust compared to ground-idle (*p* = 0.0160).

The expression of the oxidative stress marker heme oxygenase 1 (HMOX-1) was significantly up-regulated in cells exposed to Jet A-1 nvPM at ground-idle conditions (Fig. 5b; *p* = 0.0014). Interestingly, there was no statistically significant up-regulation of this gene in response to HEFA blend exposure for both thrust levels. Furthermore, expression of HMOX-1 remained low in particle-free controls for both fuel types (Fig. 5b).

As shown in Fig. 5c, there was no difference in the secretion of the pro-inflammatory cytokine interleukin-6 (IL-6) between cells exposed to nvPM of the two fuel types and engine thrust conditions or to particle-free exhaust. However, the secretion of IL-8, a chemo-attractant for neutrophilic granulocytes, was significantly increased in HEFA blend-exposed cells at 85% thrust (Fig. 5c, *p* = 0.0231). Finally, the secretion of the monocyte chemotactic protein 1 (MCP-1) was significantly higher in cell cultures exposed to nvPM for HEFA blend than Jet A-1 at ground-idle (Fig. 5c, *p* < 0.0001) and significantly higher than in cells exposed at 85% thrust with the same fuel (Fig. 5c, *p* < 0.0001).

### Morphology and reactivity of nvPM

**Fig. 6:**
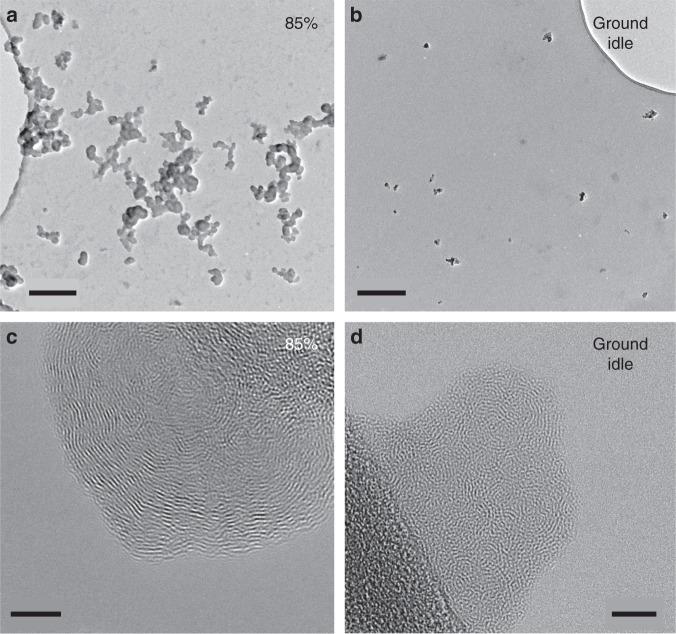
Transmission electron microscopy (TEM) images depicting morphological differences of particles with respect to size and internal structure, reflecting different reactivity. **a**, **b** Low resolution TEM images of soot particles from HEFA blend exhaust. **a** At 85% thrust. **b** At ground idle. **c**, **d** High-resolution TEM (HRTEM) images from Jet A-1 exhaust. **c** Single particle at 85% thrust. **d** Agglomerate of a few single particles at ground-idle. Images are representative for both fuel types. Scale bars: **a**, **b**, 200 nm; **c**, **d**, 5 nm

The particles examined here by TEM were primarily soot, which is the dominant nvPM besides minor amounts of ash originating from lubrication oil or fuel additives. Rarely, we observed a few, nanometer-sized ash particles attached to or enclosed in soot. Sulfur particle cores were not detected. Low magnification TEM analysis revealed considerably smaller agglomerates (maximum length, as measured on TEM images; ~300 agglomerates measured) for soot collected at ground-idle than for 85% thrust from both fuel types (Fig. 6a, b). At ground-idle, ~75% of the agglomerates from both fuel types were ≤40 nm, whereas at 85% thrust, ~50% of agglomerates were 40–120 nm. The same applied for the primary particles, which compose these agglomerates (~350 particles measured). At ground-idle, ~75% of the particles were 5–10 nm in diameter, while at 85% thrust ~70% of them were 10–25 nm, for both fuel types. These observations are consistent with previous studies describing primary soot particle sizes at taxiing and take-off conditions^18^. High-resolution TEM (HRTEM) imaging of primary soot particles from both thrust conditions and fuel types showed that they are composed of discontinuous carbon layers (fringes; Fig. 6c, d) interrupted by amorphous parts (gray areas) probably representing organic compounds, which were intermittently incorporated in the particle during its growth. It is widely accepted that the carbon lamellae of soot particles consist of more or less strongly deformed graphene sheets. The length of the carbon lamellae, as well as their orientation and the resulting degree of structural order relate to soot reactivity^34,44^. In the present study, soot particles formed at 85% thrust with both fuel types consisted of longer and better-ordered carbon lamellae (Fig. 6c), compared to those at ground-idle (Fig. 6d). Shorter carbon lamellae and a more disordered structure imply that more edge atoms are available for reaction and indicate higher instability, that is, higher reactivity. A small fraction (nearly 30%) of HEFA blend soot particles produced at 85% thrust had an outer shell, a few nanometers thick, with highly disordered fringe arrangement implying higher reactivity and indicating that at 85% thrust, a part of the HEFA blend soot is more reactive than that of Jet A-1.

## Discussion

The unique combination of the aircraft turbine emission sampling system and cell exposure experiments provides the most realistic laboratory depiction of real-life situations at the airport. This approach integrates genuine aerosol generation from a commercial aircraft turbine engine, operating with conventional Jet A-1 fuel or an alternative HEFA blend at realistic and health-relevant thrust levels, exhaust sampling directly from the emission source, and deposition of particles out of a continuous airflow onto cell cultures under physiological conditions. The electrometer data collected within the NACIVT chamber revealed successful deposition of particles onto cell cultures, and the resulting voltages were clearly discernible between the two thrust levels sampled and particle-free, that is, filtered, emissions. Moreover, changes in particle size, induced by different engine thrusts and fuels, could be observed in online measurements with a scanning mobility particle sizer (SMPS), as well as in particles evaluated by TEM analysis. The SMPS measurements are in line with the observed TEM results. This indicates that no particle aggregation occurred prior to deposition within the NACIVT chamber, which is consistent with our previous findings for 200-nm polystyrene and 20-nm silver nanoparticles^38^. Thus, the applied techniques and methodology provide a solid basis for future studies to evaluate human health hazards from exposure to aircraft turbine engine emissions.

In this study, we evaluated the adverse effects after exposure to nvPM in BEAS-2B cells, an immortalized human bronchial epithelial cell line often used in PM exposure studies^45–50^. The bronchial epithelial cell model is extremely relevant for such studies, since smaller particles deposit with high efficiency in the air-conducting section of the respiratory tract upon inhalation^24^. In fact, the highest density of intrapulmonary-deposited nanoparticles of this size per surface area is reached in the conducting airways (Supplementary Table 1^51–53^). Studying adverse effects of inhaled air pollutants to this lung compartment is important, since most lung diseases are diseases of the conducting airways, with recurrent and sustained airway inflammation being a hallmark of chronic pulmonary diseases [e.g., asthma, chronic obstructive pulmonary disease (COPD), cystic fibrosis]. Furthermore, persons with pre-existing lung disease are especially vulnerable to exposure to air pollution^27^. One-hour exposures of BEAS-2B cells to jet turbine nvPM resulted in significantly higher cytotoxicity with Jet A-1 at ground-idle (*p* = 0.0420) and HEFA blend at 85% thrust (*p* = 0.0309) compared to particle-free controls. The highest cytotoxicity and oxidative stress responses were observed after exposure to nvPM from Jet A-1 at ground-idle. This correlates with the number of deposited particles, which was 3.5-fold higher for Jet A-1 than for HEFA blend at this thrust condition. It also correlates with the small particle size and the highly disordered internal structure of Jet A-1 ground-idle soot observed by TEM, which implies higher reactivity compared to the larger sized and more ordered structure of 85% soot. Although small size and disorganized soot structure in ground-idle samples can be observed for both fuel types, Jet A-1 causes higher cytotoxicity than HEFA blend, which implies that the fuel type plays an additional role to morphology. Neither cytotoxicity nor oxidative stress responses correlate with PM mass, since the highest mass deposition is observed at 85% thrust for both fuel types. However, the observed differences in cytotoxicity and oxidative stress responses between cells exposed to nvPM from Jet A-1 and HEFA blend may be due to the lower total aromatic content in the HEFA blend. Further experimental studies are needed to elucidate which chemical components of nvPM contribute most to the observed adverse effects. While increases in cytotoxicity and oxidative stress after exposure to Jet A-1 indicate a more local effect on the epithelium, the increased release of (pro-)inflammatory cytokines after HEFA blend exposure suggests that nvPM from the latter primarily elicits a systemic effect by recruiting immune cells, that is, neutrophilic granulocytes and monocytes to the site of deposited particles in the airways. Highlighting the results and relevance of the current study, samples collected at Los Angeles’ LAX airport by He et al.^54^ were found to be more potent than samples collected in areas heavily affected by road traffic, even at low exposure concentrations.

**Fig. 7:**
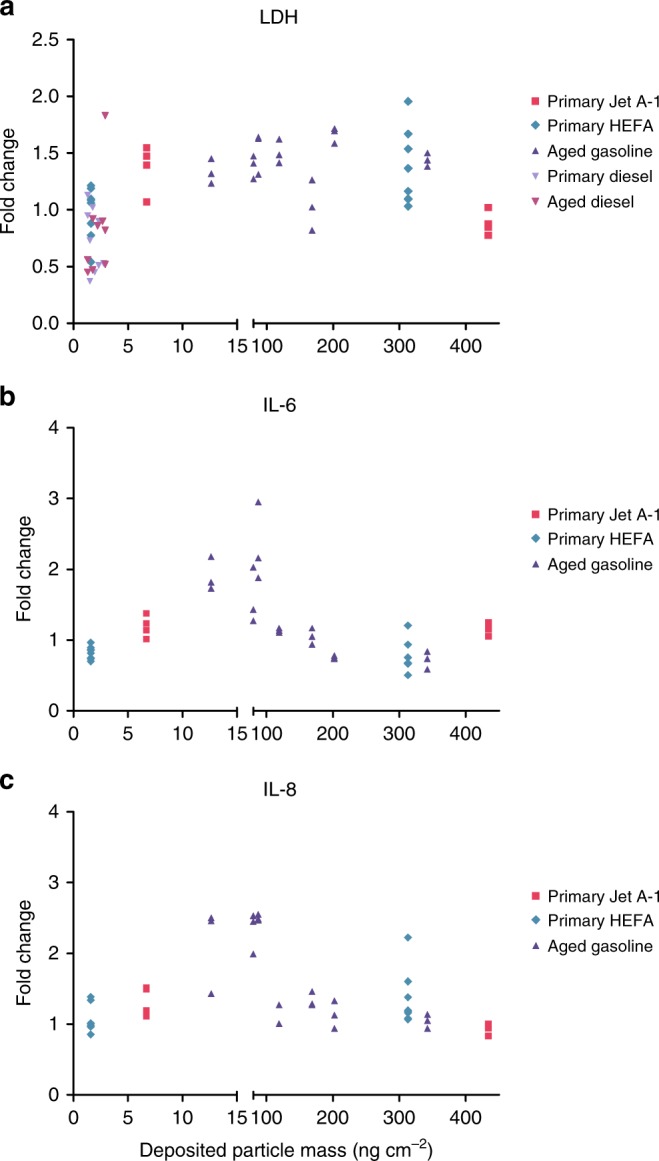
Comparison of cell responses upon exposure to gasoline, diesel, and aircraft combustion particles. **a** Cytotoxicity, **b** interleukin-6 (IL-6), and **c** IL-8 release at 24 h after exposure to diesel (data from ref. ^7^), gasoline (data from ref. ^58^), and aviation fuel particles, presented as a function of the particle dose deposited on BEAS-2B cells. Data are presented as fold change of particle-exposed cells compared to their corresponding controls, which were exposed to particle-free air (*n* = 2–8 cultures)

The composition of combustion-derived (nano)particles from different sources and conditions is highly variable^55–57^, but previous exposure experiments conducted with primary and atmospherically aged particles from gasoline and diesel engines, using a similar experimental setup and comparable deposited particle doses^8,58^, allow comparison of the toxicity of combustion-derived PM from different sources to bronchial epithelial cells (Fig. 7). This analysis shows that the cytotoxic effects of a single exposure of 1–2 h to PM from combustion of gasoline, diesel, and aviation fuel in BEAS-2B cells are moderate and comparable for similar doses. A similar pattern is observed for the secretion of IL-6 and IL-8 into basal media after exposure to gasoline and aviation fuel PM.

This study establishes, for the first time, a relationship between cellular response (effect) and morphological characteristics (likely cause) of nvPM from aircraft turbine engines. We hypothesize a primary causative link between cellular response, nvPM morphology, and fuel composition, while deposited number and mass, although critical, do not seem to be the primary factors driving the cellular responses assessed in the present study. Further research is necessary to elucidate the impact of aviation PM on human health. Moreover, Cassee and colleagues^59^ published a comprehensive review in 2013, discussing the importance of non-mass-related metrics, further strengthening our findings. We also propose differential mechanisms of how nvPM from Jet A-1 and HEFA blend fuels impair respiratory health. Well-controlled laboratory studies mimicking the in vivo situation to the greatest extent possible are warranted to better constrain adverse health effects and protect the people working at or living close to airports, especially in view of the projected growth of air traffic and the concomitant increased exposure to PM from aircraft turbine engines.

The in vitro study presented here shows that a single, short-term (1 h) exposure to nvPM from a CFM56-7B26 aircraft turbine increases cell membrane damage, leads to oxidative stress, and affects the pro-inflammatory response in bronchial epithelial cells both in a thrust level and fuel-type-dependent manner. Thus, we can expect nvPM from aircraft turbine exhaust to not only affect respiratory health but also render airway epithelia more vulnerable to secondary exposure to air pollutants and pathogens. Therefore, it is of extreme importance to characterize the exact effects of nvPM from aircraft on human health in order to protect at-risk populations. To further benefit from the unique experimental setup used in the present study, primary human airway cells should be included in any future work, as they bear functional similarity to the respiratory epithelium in vivo, most prominently mucus secretion and ciliary beating. Furthermore, cells isolated from human donors with different airway diseases like asthma and COPD, or with history of smoking, enable the study of various sub-populations and their susceptibility to adverse health effects after exhaust exposure.

## Methods

### Summary of experimental procedure

Aerosol was generated by a CFM56-7B26 turbofan engine and sampled with a standardized sampling system. Two different thrust conditions were sampled, 85% and ground-idle thrust, along with filtered aerosol as particle-free control (at 60% thrust for HEFA blend and 50% for Jet A-1). Two different fuel types were tested in the current study, conventional Jet A-1 aviation fuel and 32% v v^−1^ hydroprocessed esters and fatty acids (HEFA) blend fuel. Cultures of the human bronchial epithelial cell line BEAS-2B were exposed to the nvPM at ALI in the aerosol deposition chamber NACIVT for 60 min at physiological conditions. Relevant biomarkers of pulmonary response were measured at 24 h after exposure. Cytotoxicity was assessed by apical release of cytosolic LDH from damaged cells; oxidative stress by HMOX-1 gene expression studies and inflammatory responses by quantifying basolateral release of IL-6, IL-8, and MCP-1.

### Aerosol generation, sampling, and characterization

Combustion aerosol was sampled from a run-in, airworthy CFM56-7B26 turbofan engine running in the test cell of SR Technics at Zurich Airport. The test cell is open to the ambient environment and the engine was fueled with standard Jet A-1 fuel (one exposure day) or a 32% v v^−1^ HEFA fuel blend (two separate exposure days). Fuel specifications are provided in Table 2. Engine thrust levels were controlled using the engine combustor inlet temperature (T3), for which the corresponding thrust levels for international standard atmospheric conditions (15 °C, 1013.25 hPa) are known. This approach is ordinarily used for the environmental certification of aircraft engines and in scientific studies^29,60^. Engine exhaust was collected using a state-of-the-art sampling system (Fig. 1a), which complied with the recommended standardized sampling practice^61^. The Multi Channel Process System (MCPS 7.0, CAD computer Inc.) software suite was used to collect real-time PM instrumentation data with a 1-Hz resolution and real-time data processing (flagging of stable sampling periods, merging of files) was performed in MATLAB R2017 (Mathworks Inc.) with a custom code. The NACIVT chamber was connected to the diluted PM sampling line in parallel to the particle instrumentation, which consisted of a Micro Soot Sensor (MSS, Model 483, AVL Inc.) for photo acoustically measuring particle mass^62^, an AVL particle counter (APC, Model 489, AVL Inc.) to determine particle number^63^, and a SMPS (Model 3938 consisting of the long differential mobility analyzer Model 3081A and the condensation particle counter Model 3776, TSI Inc.) to measure particle size distributions. The lower particle size cut-offs were 10 nm for the APC and 6 nm for the SMPS measurements. Particle size distribution data from the SMPS was collected using Aerosol Instrument Manager (AIM 10.2, TSI Inc.). Volatile organic compounds were removed upstream of the NACIVT chamber with a custom-made low-flow thermodenuder^64^ operated at 200, 200, and 100 °C on the preconditioning, first absorption, and second absorption sections, respectively. The particle data reported here correspond to conditions at the inlet of the exposure chamber. The actual exhaust concentrations at the engine exit are substantially higher due to sampling system losses and dilution (∼1:10). The sampling losses from the engine exit plane to the NACIVT inlet were evaluated similar to Durdina et al.^60^ using the measured SMPS particle size distributions and the size-dependent sampling line penetration functions, which consider thermophoretic and diffusional particle losses. Particle size integrated loss correction factors from the NACIVT inlet to the engine exit plane were in the range of 1.31–1.42 for particle mass and 2.45–5.96 for particle number, with the highest losses associated with the smallest particles at engine ground-idle. These correction factors account for differences in particle line losses between the PM instrumentation inlets and the NACIVT inlet, which were found to be in the range of 3–7% for non-volatile particle mass and 15–40% for non-volatile particle number^65^. All PM data was plotted using Igor Pro 7.0 (Wavemetrics Inc.) and Origin 2017 (Originlab Inc).

### Cell cultures and aerosol exposures

Submersed, low passage BEAS-2B cells (CRL-9609™, American Type Culture Collection ATCC^®^, LGC Standards sàrl, Molsheim, France) were maintained in 10-cm PRIMARIA™ Tissue Culture Dishes (BD Falcon™, BD Biosciences, Allschwil, Switzerland) in 12 mL of serum-free LHC-basal medium (Gibco, Thermo Fisher Scientific, Life Technologies Europe B.V., Zug, Switzerland) supplemented with growth factors suitable for bronchial epithelial cell growth as previously described^66^. For aerosol exposure, cells were seeded at the density of 2 × 10^5^ cells cm^−2^ for next day exposure on microporous 6.5 mm Transwell^®^ supports (polyester membrane, 0.4 µm pores; Corning^®^) previously coated with type IV collagen (Merck & Cie, Schaffhausen, Switzerland), in recommended volume of medium containing retinoic acid in apical medium only. Four to eight hours prior to aerosol exposure, the apical surface of cells was washed once with Dulbecco’s phosphate-buffered saline (Gibco), basolateral media exchanged, and apical medium reduced to minimum (<1 mm, 40 µL) to mimic ALI conditions.

In the NACIVT chamber, the aerosol was applied to BEAS-2B cell cultures for 60 min, directly out of a conditioned airflow at physiological conditions. A fixed exposure duration was chosen for all thrust levels to have the same particle retention and clearance conditions in all cell cultures. Furthermore, particle doses deposited on the cells were comparable to our previous study^8,58^ and corresponded to daily doses delivered to the human tracheobronchial area at an ambient pollution level of <10 and 500 µg m^−3^ (for calculations see Supplementary Table 2). The chamber has been described in detail previously^23^. Briefly, the airstream passes a low-flow thermodenuder upstream of the NACIVT removing gaseous organic species^64^, and a unipolar diffusion charger applying 1–4 positive net charges depending on the mobility diameter. Thereafter, the aerosol flow is split into two fractions. One fraction is drawn to an internal aerosol electrometer to measure the particle concentration for the deposited particle-dose estimates on cells. The other fraction is conditioned to the physiological environment of mammalian cells, that is, to 37 ± 1 °C, 85 ± 5% relative humidity, and 5 ± 2% CO_2_^38,67,68^, and passed on to the cell exposure chamber, where it is equally divided into 24 delivery tubes at a resulting flow rate of 25 mL min^−1^ per Transwell^®^ insert. Precipitation of particles on cells occurs by electrostatic deposition, applying a DC voltage of 2 kV between the end of each aerosol delivery tube and the insert-holder plate. This results in uniform and efficient deposition of sub-micrometer-sized particles, reaching target tissue doses comparable to human ambient exposures^69^. For particle-free exposure, a Balston DFU Model 9933-11, grade BQ filter (Parker Hannifin Corporation, New York, USA) was mounted between the aerosol exhaust line and the thermodenuder. To account for the effects of transport, a non-treated incubator control was kept in parallel but never subjected to any exposure treatment in the NACIVT exposure chamber. Real-time particle deposition was estimated with Lab View 9.0.1.

### Cell responses

Relevant biological markers indicating acute toxicity and impairment of normal lung function were selected based on previous studies with combustion-derived aerosol^8,58^ and measured 24 h after exposure to aerosol. Cytotoxicity was evaluated by the release of cytosolic LDH from damaged cells. Apical washes were collected 4 and 24 h post exposure and stored at −20 °C until analysis using the colorimetric cytotoxicity detection kit^PLUS^ (Roche Diagnostics AG, Rotkreuz, Switzerland) according to the manufacturer’s instructions. Data were collected using EL808 microplate reader (BioTek Instruments GmbH, Sursee, Switzerland) and the corresponding software (KC Junior). Maximum releasable LDH was estimated in the supernatants of cells lysed with 100 µL 1% Triton-X solution for 10 min at 37 °C. Prior to toxicity calculations, absorption values were adjusted for volume differences. Cytotoxicity is presented as fold change in LDH activity (absorbance) over incubator control. The expression of the oxidative stress marker HMOX-1 was detected by quantitative polymerase chain reaction (qPCR). Cells were lysed in TRIzol^®^ (Thermo Fisher Scientific) and total cell RNA extracted using Zymo Research Direct-zol^TM^ mini prep plus columns (Lucerna Chem, Luzern, Switzerland) according to the manufacturer’s instructions. The complementaary DNA (cDNA) was prepared using the QuantiTect^®^ reverse transcription kit (Qiagen, Hombrechtikon, Switzerland) according to the manufacturer’s instructions. Briefly, genomic DNA (gDNA) was removed from RNA samples with the provided gDNA Wipeout buffer for 2 min at 42 °C and subsequently stored on ice. Reverse transcription (RT) was performed with the provided RT mixes for 15 min at 42 °C, 3 min at 95 °C to inactivate enzymes, and stored at −20 °C until qPCR analysis. For gene expression analysis, 2 μL of tenfold diluted total cDNA was amplified using the Applied Biosystems 7900HT system (Thermo Fisher Scientific). The cycling parameters were: 15 min at 95 °C, 45 cycles of 15 s at 94 °C, 30 s at 55 °C, and 30 s at 72 °C, followed by a dissociation step confirming product specificity. Biological replicates were analyzed three separate times. Data were analyzed using Applied Biosystems SDS v2.4 and normalized to hypoxanthine-guanine phosphoribosyltransferase. Data are presented as fold change over incubator control. Release of the inflammatory mediators IL-6, IL-8, and MCP-1 into basolateral media was assessed 24 h after aerosol exposure. Samples were collected and stored at −20 °C until analysis. Cytokine secretions were quantified using the BioPlex-Pro™ 27-plex assay (Bio-Rad Laboratories AG, Cressier, Switzerland) and the corresponding software (BioPlex Manager 6.1) according to the manufacturer’s instructions and are represented as absolute quantification (pg mL^−1^).

### Transmission electron microscopy (TEM) of soot particles

The particles studied by TEM were collected onto Cu-supported holey carbon film grids (200 mesh) inside the NACIVT chamber for 60 min, in parallel with cell exposures. TEM imaging was performed with a JEOL 2200FS microscope equipped with an Omega filter, a Schottky field emission gun at 200 kV, and a point-to-point resolution of 0.23 nm. Images were taken in low-resolution (TEM) and high-resolution (HRTEM) mode. Gatan DigitalMicrograph^®^ was used for image analysis.

### Statistical analysis

Cell cultures were exposed to aerosols in quadruplicates. Biological data are presented as fold change over incubator control or absolute quantification (pg mL^−1^). Statistical significance was determined with GraphPad Prism (v5 and v7) using a non-matching two-way analysis of variance with Bonferroni post-tests. Results are considered as statistically significant for *p* values < 0.05.

### Reporting summary

Further information on experimental design is available in the Nature Research Reporting Summary linked to this article.

## Supplementary information

Supplemental Information

Reporting Summary

## Data Availability

All relevant source data are available from the authors. The following Figures and Tables have associated source data: Fig. 1b–d; Fig. 2b; Fig. 5a–c; Fig. 7a–c; Table 2.
