# Supplementary material for: Non-volatile particle emissions from aircraft turbine engines at ground-idle induce oxidative stress in bronchial cells
Source: Commun Biol. 2019 Mar 5;2:90. doi: 10.1038/s42003-019-0332-7 (PMC6401161; doi:10.1038/s42003-019-0332-7)
Supplement: Supplementary file 1 — Supplemental Information [file 42003_2019_332_MOESM1_ESM.pdf]

Supplementary Information: Non-volatile particle emissions from aircraft turbine engines at ground-idle induce oxidative stress in bronchial cells

Supplementary Figures

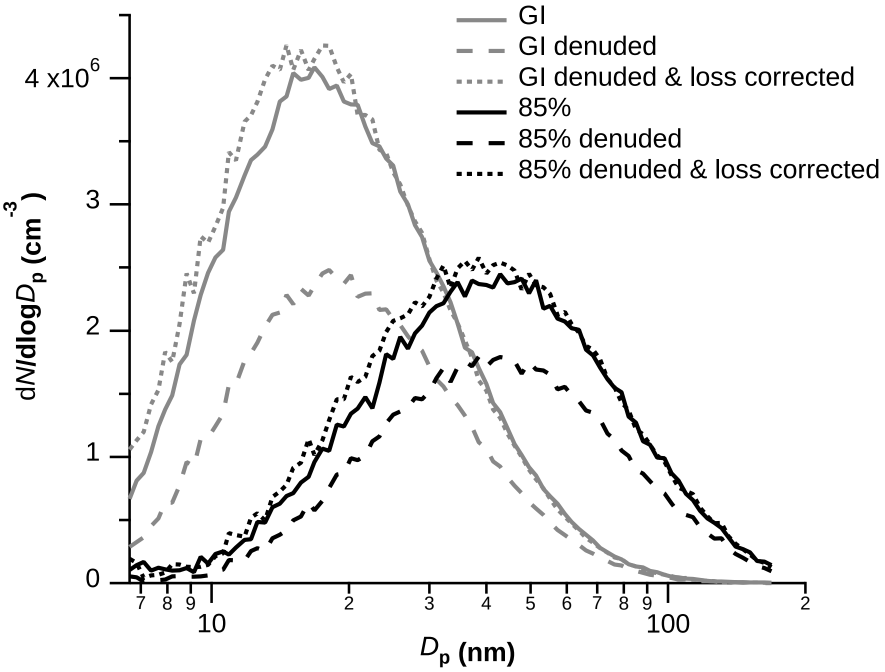

Supplementary Figure 1. Undenuded - (solid lines), denuded- (dashed lines) and denuded, particle loss corrected- (dotted lines) size distributions for the ground-idle and 85% engine thrust conditions. Data were collected with the same engine model (with a different engine serial number) on a separate measurement day burning Jet A-1 fuel.

Supplementary Tables

Supplementary Table 1 – Estimates for nvPM deposition in human lungs*

	Ground-idle				85% thrust			
	HEFA blend		Jet-A1		HEFA blend		Jet-A1	
**Particle size, diameter** [nm]	17	17	18	18	47	47	50	50
**Aerosol concentration** [cm^{-3}]	1.00E+05	1.00E+06	1.00E+05	1.00E+06	1.00E+05	1.00E+06	1.00E+05	1.00E+06
**Aerosol concentration** [m^{-3}]	1.00E+11	1.00E+12	1.00E+11	1.00E+12	1.00E+11	1.00E+12	1.00E+11	1.00E+12
**Inhaled air volume, 1 h, adult** [m^{3}]	0.45	0.45	0.45	0.45	0.45	0.45	0.45	0.45
**Inhaled air volume, 24 h, adult** [m^{3}]	10.8	10.8	10.8	10.8	10.8	10.8	10.8	10.8
**Inhaled particles, 1 h**	4.5E+10	4.5E+11	4.5E+10	4.5E+11	4.5E+10	4.5E+11	4.5E+10	4.5E+11
**Inhaled particles, 24 h**	1.08E+12	1.08E+13	1.08E+12	1.08E+13	1.08E+12	1.08E+13	1.08E+12	1.08E+13
**Total deposited fraction**	0.747	0.747	0.738	0.738	0.526	0.526	0.510	0.510
**Deposited particles, 1 h**	3.36E+10	3.36E+11	3.32E+10	3.32E+11	2.37E+10	2.37E+11	2.29E+10	2.29E+11
**Deposited particles, 24 h**	8.07E+11	8.07E+12	7.97E+11	7.97E+12	5.68E+11	5.68E+12	5.50E+11	5.50E+12
**Alveolar deposited fraction**	0.243	0.243	0.253	0.253	0.274	0.274	0.268	0.268
**Deposited particles, 1 h**	1.09E+10	1.09E+11	1.14E+10	1.14E+11	1.23E+10	1.23E+11	1.20E+10	1.20E+11
**Deposited particles, 24 h**	2.62E+11	2.62E+12	2.73E+11	2.73E+12	2.96E+11	2.96E+12	2.89E+11	2.89E+12
**Conducting airway deposited fraction**	0.360	0.360	0.348	0.348	0.187	0.187	0.180	0.180
**Deposited particles, 1 h**	1.62E+10	1.62E+11	1.57E+10	1.57E+11	8.42E+09	8.42E+10	8.10E+09	8.10E+10
**Deposited particles, 24 h**	3.89E+11	3.89E+12	3.76E+11	3.76E+12	2.02E+11	2.02E+12	1.94E+11	1.94E+12
**Alveolar surface area** [m^{2}]	140	140	140	140	140	140	140	140
**Particles per surface area, 1 h** [cm^{-2}]	7.811E+03	7.811E+04	8.132E+03	8.132E+04	8.814E+03	8.814E+04	8.598E+03	8.598E+04
**Particles per surface area, 24 h** [cm^{-2}]	1.875E+05	1.875E+06	1.952E+05	1.952E+06	2.115E+05	2.115E+06	2.064E+05	2.064E+06
**Conducting airways surface area** [m^{2}]	2	2	2	2	2	2	2	2
**Particles per surface area, 1 h** [cm^{-2}]	8.109E+05	8.109E+06	7.832E+05	7.832E+06	4.208E+05	4.208E+06	4.048E+05	4.048E+06
**Particles per surface area, 24 h** [cm^{-2}]	1.946E+07	1.946E+08	1.880E+07	1.880E+08	1.010E+07	1.010E+08	9.715E+06	9.715E+07

*Multiple-Path Particle Dosimetry Model (MPPD v. 3.04. 2018): A Model for Human and Rat Airway Particle Dosimetry (50-52).

Supplementary Table 2 – Particle dose in human tracheobronchial (TB) tract at different ambient concentrations*

		Ground-idle		85% thrust	
		**HEFA blend**	**Jet-A1**	**HEFA blend**	**Jet-A1**
**Particle size, diameter** [nm]		17	18	47	50
**Particle density** [g cm^{-3}]		1.00	1.00	1.00	1.00
**Tidal Volume**, *V*T [m^3][(1)]		0.000625	0.000625	0.000625	0.000625
**Breathing frequency**, *f* [min^{-1}]		12	12	12	12
**Inhaled air volume, 1 h, adult** [m^3]		0.45	0.45	0.45	0.45
**Inhaled air volume, 24 h, adult** [m^3]		10.8	10.8	10.8	10.8
**Surface area TB tract** [cm^2]		2471	2471	2471	2471
**Deposition fraction***		0.360	0.348	0.187	0.180
**Ambient mass concentration** [mg m^{-3}]	10				
**Mass per surface area TB, 24 h** [ng cm^{-2}]		16	15	8	8
**Ambient mass concentration** [mg m^{-3}]	20				
**Mass per surface area TB, 24 h** [ng cm^{-2}]		32	30	16	16
**Ambient mass concentration** [mg m^{-3}]	100				
**Mass per surface area TB, 24 h** [ng cm^{-2}]		158	152	82	79
**Ambient mass concentration** [mg m^{-3}]	200				
**Mass per surface area TB, 24 h** [ng cm^{-2}]		315	304	163	157
**Ambient mass concentration** [mg m^{-3}]	400				
**Mass per surface area TB, 24 h** [ng cm^{-2}]		630	609	327	315
**Ambient mass concentration** [mg m^{-3}]	500				
**Mass per surface area TB, 24 h** [ng cm^{-2}]		788	761	409	393
**Ambient mass concentration** [mg m^{-3}]	600				
**Mass per surface area TB, 24 h** [ng cm^{-2}]		945	913	490	472

*Model used for calculations: Multiple Path Particle Dosimetry Model (Multiple-Path Particle Dosimetry Model (MPPD v. 3.04. 2018. nchial: A Model for Human and Rat Airway Particle Dosimetry (50-52).
