# Supplementary material for: Non-volatile particle emissions from aircraft turbine engines at ground-idle induce oxidative stress in bronchial cells
Source: Commun Biol. 2019 Mar 5;2:90. doi: 10.1038/s42003-019-0332-7 (PMC6401161; doi:10.1038/s42003-019-0332-7)
Supplement: Supplementary file 2 — Reporting Summary [file 42003_2019_332_MOESM2_ESM.pdf]

nature research

Corresponding author(s): Marianne Geiser, Anthi Liati, Benjamin Brem

Last updated by author(s): 2019/01/10

Reporting Summary

Nature Research wishes to improve the reproducibility of the work that we publish. This form provides structure for consistency and transparency in reporting. For further information on Nature Research policies, see Authors & Referees and the Editorial Policy Checklist.

Statistics

For all statistical analyses, confirm that the following items are present in the figure legend, table legend, main text, or Methods section.

n/a	Confirmed	
☐	☒	The exact sample size (n) for each experimental group/condition, given as a discrete number and unit of measurement
☐	☒	A statement on whether measurements were taken from distinct samples or whether the same sample was measured repeatedly
☐	☒	The statistical test(s) used AND whether they are one- or two-sided<br>*Only common tests should be described solely by name; describe more complex techniques in the Methods section.*
☐	☒	A description of all covariates tested
☐	☒	A description of any assumptions or corrections, such as tests of normality and adjustment for multiple comparisons
☐	☒	A full description of the statistical parameters including central tendency (e.g. means) or other basic estimates (e.g. regression coefficient) AND variation (e.g. standard deviation) or associated estimates of uncertainty (e.g. confidence intervals)
☒	☐	For null hypothesis testing, the test statistic (e.g. F, t, r) with confidence intervals, effect sizes, degrees of freedom and P value noted<br>*Give P values as exact values whenever suitable.*
☒	☐	For Bayesian analysis, information on the choice of priors and Markov chain Monte Carlo settings
☒	☐	For hierarchical and complex designs, identification of the appropriate level for tests and full reporting of outcomes
☒	☐	Estimates of effect sizes (e.g. Cohen's d, Pearson's r), indicating how they were calculated

Our web collection on statistics for biologists contains articles on many of the points above.

Software and code

Policy information about availability of computer code

Data collection	Lab View 9.0.1, Applied Biosystems SDS v2.4, Bio-Plex Manager 6.1, KC Junior, MCPS7, TSI AIM, Gatan DigitalMicrograph
Data analysis	Graph Pad Prism v5 and v7, Applied Biosystems SDS v2.4, Microsoft Excel 2016, MATLAB R2017b, Igor Pro, Origin 2017

For manuscripts utilizing custom algorithms or software that are central to the research but not yet described in published literature, software must be made available to editors/reviewers. We strongly encourage code deposition in a community repository (e.g. GitHub). See the Nature Research guidelines for submitting code & software for further information.

Data

Policy information about availability of data

All manuscripts must include a data availability statement. This statement should provide the following information, where applicable:

- Accession codes, unique identifiers, or web links for publicly available datasets
- A list of figures that have associated raw data
- A description of any restrictions on data availability

All source data is available from corresponding authors upon request

Field-specific reporting

Please select the one below that is the best fit for your research. If you are not sure, read the appropriate sections before making your selection.

☒ Life sciences ☐ Behavioural & social sciences ☐ Ecological, evolutionary & environmental sciences

For a reference copy of the document with all sections, see nature.com/documents/nr-reporting-summary-flat.pdf

Life sciences study design

All studies must disclose on these points even when the disclosure is negative.

Sample size	Experiments were designed using at least three biological replicates per sampling condition, since this is the minimum for any inferential analysis. The real time particle data sample size was 3600 per sampling condition (1hr collection time at a 1Hz resolution). 80 particle size distributions were collected per sampling condition.
Data exclusions	Two samples were excluded from quantitative real time PCR analysis due to poor RNA quality.
Replication	Results described in the current study were not replicated independently as a whole, i.e. experiments with Jet A-1 fuel were performed on one exposure day only, while experiments with HEFA fuel blend were performed on two separate exposure days. Furthermore, four biological replicates were analyzed for each experimental condition (thrust level). Due to extreme costs and logistical challenges, the experiments are difficult to repeat.
Randomization	Cell cultures were randomly allocated into experimental groups from a collection of 24-well cell culture plates.
Blinding	Blinding was not relevant to the current study since no human participants were involved and therefore no bias had to be reduced. Furthermore, in-vitro studies traditionally do not utilize blinding.

Reporting for specific materials, systems and methods

We require information from authors about some types of materials, experimental systems and methods used in many studies. Here, indicate whether each material, system or method listed is relevant to your study. If you are not sure if a list item applies to your research, read the appropriate section before selecting a response.

Materials & experimental systems

n/a	Involved in the study	
☐	☒	Antibodies
☐	☒	Eukaryotic cell lines
☒	☐	Palaeontology
☒	☐	Animals and other organisms
☒	☐	Human research participants
☒	☐	Clinical data

Methods

n/a	Involved in the study	
☒	☐	ChIP-seq
☒	☐	Flow cytometry
☒	☐	MRI-based neuroimaging

Antibodies

Antibodies used	Bio-Plex Pro Human Cytokine lL-6 Set #171B5006M, Bio-Plex Pro Human Cytokine IL-8 Set #171B5008M, Bio-Plex Pro Human Cytokine MCP-1 (MCAF) Set #171B5021M
Validation	The following parameters are applied by BIO RAD for product validation: Specificity (cross-reactivity), accuracy (recovery) in key sample matrices, inter- and intra-assay precision, sensitivity (limit of detection, LOD), assay working range (LLOQ/ULOQ), linearity of dilution, parallelism and matrix effect, performance characteristics in real samples. http://www.bio-rad.com/webroot/web/pdf/lsr/literature/Bulletin_6499.pdf

Eukaryotic cell lines

Policy information about cell lines

Cell line source(s)	American Type Culture Collection ATCC®, LGC Standards sàrl, Molsheim, France
Authentication	Cell line was authenticated by microscopic morphological evaluation
Mycoplasma contamination	Cell line was not tested for mycoplasma contamination
Commonly misidentified lines (See ICLAC register)	No commonly misidentified cell lines were used in this study
